# Association of Diet Texture With Denture Wearing and Denture Quality in Institutionalized Older Adults Requiring Long-Term Care

**DOI:** 10.7759/cureus.97796

**Published:** 2025-11-25

**Authors:** Mana Hirayama, Yukiko Hatanaka, Kunihito Yamane, Tomoko Mukai, Kae Namikawa, Masatsugu Teraoka, Tomohiro Tabata, Housei Suzuki, Kazuki Ako, Junichi Furuya

**Affiliations:** 1 Oral Function Management, Graduate School of Dentistry, Showa Medical University, Tokyo, JPN

**Keywords:** denture quality, diet texture, dysphagia, long-term care, older adults, oral function, oral health

## Abstract

Background

To maintain and enhance the enjoyment of meals among older adults in long-term care, and to improve malnutrition and prevent aspiration pneumonia, it is essential to evaluate and train their swallowing function, as well as provide dental interventions. Delivering high-quality dentures to that population would support mastication and swallowing, which would aid in achieving this goal; however, the relationship between the denture quality and diet texture remains unclear.

Objective

The purpose of this study was to elucidate the association between high diet texture and the use of high denture quality by evaluating diet texture, denture use, and objective denture quality among institutionalized older adults.

Methods

A cross-sectional survey was conducted among 197 older adults requiring nursing care who had been newly admitted (within a few months of study initiation) to specific older adult care facilities and regularly consumed an oral diet. Age, care-need level, diet texture, swallowing function, number of remaining teeth, oral health status, and denture use were also investigated. For 81 denture wearers, denture quality was assessed using a 5-Defects score.

Results

Multiple regression analysis revealed that diet texture decreased with increasing age and care needs. Conversely, good swallowing function, a higher number of remaining teeth, and denture use were associated with a higher level of diet texture. Furthermore, among denture wearers, higher denture quality was associated with a higher level of diet texture.

Conclusion

Higher diet texture among care-dependent older adults was significantly associated not only with denture use but also with the quality of their dentures. Wearing high-quality dentures can improve masticatory and swallowing functions, leading to more efficient nutrient intake and greater enjoyment of eating through increased dietary variety and improved diet texture. As denture use and quality can be improved through dental intervention, this supports the importance of prosthodontic approaches in promoting the enjoyment of meals among care-dependent older adults.

## Introduction

As life expectancy increases and the global population ages due to advances in medical technology, Japan - the most advanced super-aged society - faces major social concerns arising from the growing number of older adults requiring long-term care and the prolonged duration of care associated with extended life expectancy [1]. The primary causes of long-term care dependency-such as dementia, cerebrovascular disease, and Parkinson’s disease-often impair oral and swallowing functions, leading to loss of the ability to enjoy eating and a decline in quality of life (QOL) [2,3]. Among institutionalized older adults, cognitive and physical impairments frequently hinder the implementation of dysphagia rehabilitation. Thus, compensatory strategies, such as diet texture modifications tailored to individual functional capacities, represent practical and effective approaches for nutritional support [4].

Texture-modified diets (e.g., minced or pureed foods) are commonly prescribed to institutionalized residents. Swallowing function, nutritional status, activities of daily living (ADL), cognitive ability, denture use, and oral health status have been shown to influence diet texture [5]. Since eating is a central source of enjoyment in daily life, appetite and satisfaction with food are closely linked to nutritional status [6,7]. Therefore, supporting food intake is critical to prevent malnutrition and maintain the QOL. Optimization of oral functions, including tongue function and denture use, is essential to maximize swallowing potential and preserve or improve diet texture whenever possible [8].

Older adults who require long-term care often experience tooth loss and a high need for dentures; however, nonuse and ill-fitting dentures remain common, contributing to impaired mastication and swallowing function, and nutritional status [9,10]. Properly fitted dentures restore dentition and jaw structure, stabilize oropharyngeal movements, enhance bolus transport during mastication and swallowing, support the oral preparatory and oral stages, and improve the safety of the pharyngeal stage [11,12].

Consequently, nonuse of dentures or poor denture quality may result in reduced oral intake and restricted diet texture [13]. Previous studies have linked denture use to maintaining a higher level of diet texture [8,10,13]. However, they have focused only on denture use rather than denture quality. Although many older adults wear poor-quality dentures, and dental interventions can improve their quality, the relationship between denture quality and diet texture remains unclear. We hypothesized that, in addition to denture use, the quality of dentures would be independently associated with diet texture.

Thus, this study aimed to elucidate the association between high diet texture and the use of high quality denture by evaluating diet texture, denture use, objective denture quality, and other confounding factors among institutionalized older adults.

## Materials and methods

Participants

The participants in this study were 307 older adult individuals requiring nursing care (65 males, 242 females; mean age, 86.6 ± 8.3 years). The inclusion criteria were older adults aged 65 years and over who were newly admitted to two long-term care facilities in Tokyo, where we regularly provide home-visit dentistry, between August 2020 and March 2024. The exclusion criteria were as follows: (1) primary nutrition intake method was non-oral, such as tube feeding; (2) missing data in the assessment items; and (3) individuals who declined participation through the opt-out process. A total of 46 participants were excluded because their primary nutrition intake was non-oral feeding, and 64 participants were excluded due to missing data. Finally, 197 participants were included in this analysis. As a result of the post hoc power analysis conducted using G*Power 3.1, the statistical power of the analysis was 1.0 with an alpha error of 0.05, based on the adjusted coefficient of determination.

Outcomes

In addition to age, sex, comorbidities, care-need level, dysphagia severity scale (DSS), diet texture, oral health status, number of teeth, denture use, and denture quality, the following information was retrospectively extracted from the medical and conference records.

Comorbidities

Comorbidities were assessed using the Charlson Comorbidity Index (CCI) to evaluate each subject’s systemic condition [14]. The CCI assigns weights to 19 chronic diseases and uses the total score to indicate disease burden. In this study, the presence of each disease was determined based on medical records and physician diagnoses.

Care-need level

The level of care needed is determined by the municipal governments based on physician opinions and certification surveys, resulting in an official certification classification. Care Level 1 indicated the lowest level of care needs, whereas Care Level 5 indicated the highest. Level 1 requires partial assistance with ADL and shows a slight cognitive decline. Level 2 requires more assistance with ADL than with Level 1 patients and shows noticeable cognitive decline. Level 3 requires comprehensive aid with ADL, with the individual using a cane, walker, or wheelchair to stand and walk. Cognitive function declines, and supervision is necessary. Level 4 requires assistance in all aspects of daily life, surpassing the requirements of Level 3. A significant decline in cognitive abilities, including thinking and comprehension, was also observed. Level 5 requires assistance with all ADL, and communication becomes difficult.

Dysphagia severity scale

The DSS was used to assess swallowing function [15]. The DSS is a seven-level scale that evaluates dysphagia severity based on clinical observations, such as post-swallow residue, laryngeal entry, and aspiration. A score of 7 indicates a normal range, 6 indicates mild problems, 5 indicates oral problems, 4 indicates occasional aspiration, 3 indicates aspiration of liquids, 2 indicates aspiration of food, and 1 indicates aspiration of saliva. A score of 7 was considered normal, and a score of 1 indicated the most severe level.

Diet texture

The Functional Oral Intake Scale (FOIS) was used to evaluate the daily intake of diet texture [16]. The FOIS is a 7-level ordinal scale that assesses the presence or absence of oral intake, primary route of nutritional intake, and necessity for diet texture modification or preparation. Level 1 indicates tube feeding with no oral intake; level 2 indicates tube feeding with minimal oral intake for enjoyment; level 3 indicates tube feeding as the primary method, but with ongoing oral intake; levels ≥ 4 suggest that nutritional intake is possible via oral intake alone. Level 4 represents oral nutrition, consisting solely of a single texture, such as pureed food. Level 5 involves oral nutrition, requiring special preparation or compensatory measures for multiple textures, such as chopped or soft foods. Level 6 involves oral nutrition requiring specific restrictions but no special preparation for multiple textures, such as soft-cooked rice and soft vegetables. Level 7 represents oral nutrition of regular foods with no particular restrictions. The dietary texture of participants was determined at the time of admission to the facility through a multidisciplinary conference led by physicians, dietitians, and care managers, with the involvement of dentists, nurses, and other professionals.

Oral health status

The Japanese version of the Oral Health Assessment Tool (OHAT-j) was used to comprehensively assess oral health status [17]. The OHAT was developed to evaluate the oral health of older adults from multiple perspectives. It is primarily used for screening purposes in clinical settings, such as hospitals and facilities, to facilitate professional dental intervention and evaluate oral health status in clinical research. The OHAT consists of eight items: lips, tongue, gums, oral mucosa, saliva, natural teeth, dentures, oral hygiene, and dental pain. Each item is rated on a three-point scale: “Healthy (0 points)”, “Oral changes present (1 point)”, “Unhealthy (2 points)”. The total score ranges from 0 to 16 points, with higher scores indicating poorer overall oral health.

Number of teeth, denture use, and denture quality

The number of teeth was defined as the number of remaining teeth, excluding root remnants, teeth with mobility grade 3, and dental implants. The denture use status was considered present if a denture was worn daily, even if it was only on one jaw (upper or lower). Conversely, it was considered absent if there were no edentulous areas, or edentulous areas existed but no dentures were worn. For denture wearers, denture quality was assessed using a 5-Defects system [18]. The 5-Defects system assesses the structural and functional quality of dentures based on the following five criteria: 1) structural defects, such as damage to the denture base, artificial teeth, or attachments; 2) significant wear on molar artificial teeth; 3) use of denture adhesive or presence of tissue conditioning; 4) problems with denture stability; 5) problems with denture retention. For each item, both maxillary and mandibular dentures were evaluated on a 2-point scale: 0 points (not applicable) or 1 point (applicable). Denture stability was assessed based on whether the displacement of the occlusal rests or indirect retention devices was ≤ 1 mm when force was applied to the denture base from one or both sides, or whether the denture lifted approximately 1 mm when force was applied to the stressed area. Retention was assessed by determining whether the denture was dislodged when the patient opened their mouth without exerting any strain. The 5-Defects score represents the objective denture quality on a 10-point scale, where 0 indicates good quality and 10 indicates poor quality. For patients wearing dentures in both the maxilla and the mandible, a maximum total score of 10 points was calculated. For patients wearing dentures in only one jaw, a maximum total score of 5 points was calculated and converted to a 10-point scale. Assessments of eating and swallowing function, food consistency, oral health status, number of remaining natural teeth, presence of dentures, and denture quality were performed by two dentists who had undergone training and prior calibration of the assessment criteria. Inter-rater reliability, as measured by the intraclass correlation coefficient, was 0.942.

Statistical analysis

To elucidate whether the use of higher-quality dentures could improve diet texture among dependent older adults, we performed an exploratory multiple regression analysis to examine the relationships between diet texture and denture use, as well as between diet texture and denture quality, accounting for potential confounding factors. The normality of data was confirmed using a Q-Q plot, which showed that the residuals were approximately normally distributed. The dependent variable was FOIS score, and the following two analytical models were established: Model 1 examined the relationship between diet texture and denture use in all participants (n = 197) using multiple regression analysis, with age, sex, CCI, care-need level, OHAT-j score, DSS, number of teeth, and denture use as independent variables. Model 2 restricts the analysis to denture wearers (n = 81). We added the 5-Defects score to the independent variables instead of denture use from Model 1 and performed a multiple regression analysis to examine the relationship between diet texture and denture quality. To ensure the consistency of the study, the forced-entry method was applied in the multiple regression analysis of models 1 and 2. Statistical analyses were performed using IBM SPSS Statistics v27.0 (IBM Japan, Tokyo, Japan), with the significance level set at < 5% for all tests. This study was approved by the Showa University Research Ethics Review Board Committee (Approval No.: 21-075-B) and commenced after obtaining informed consent via an opt-out method according to the ethical guidelines for medical research involving human subjects issued by the government and the STROBE (STrengthening the Reporting of OBservational studies in Epidemiology) statement.

## Results

Table 1 lists the characteristics of the 197 participants. Most participants were over 80 years old, and approximately 83% were females. The participants’ care needs were also high, and they did not have a sufficient number of teeth. Approximately 41% of the participants used dentures, with a mean denture quality (5-Defects score) of 3.3 on a 10-point scale. The results of Model 1, which analyzed the association between diet texture and denture use, and Model 2, which analyzed the association between diet texture and denture quality, are shown in Table 2 and Table 3, respectively. Even after adjusting for confounding factors, a high level of diet texture was negatively associated with age (β=-0.150) and care-need level (β=-0.186), and positively associated with DSS (β=0.638), number of teeth (β=0.110), and denture use (β=0.145). The 5-Defects score (β= −0.198), in which a higher value indicates lower denture quality, showed a negative association with the high level of diet texture.

**Table 1:** Participant characteristics CCI: Overall health status was assessed using the Charlson Comorbidity Index; Care-need level: Ranges from 1 (mildest) to 5 (most severe). DSS: Ranges from 3 (liquid aspiration level) to 7 (within normal limits). FOIS: Ranges from 4 (pureed diet) to 7 (regular diet). OHAT-j score: Ranges from 0 (best oral health status) to 16 (poorest oral health status). Denture wear was defined as the use of dentures, including cases where only one arch was fitted. 5-Defects score: Scored from 0 (best denture quality) to 10 (poorest denture quality). For participants wearing single-arch dentures, the score was first calculated on a 5-point scale and then converted to a 10-point scale. CCI: Charlson comorbidity index, DSS: dysphagia severity scale, FOIS: functional oral intake scale, OHAT-j: oral health assessment tool (Japanese version), SD: standard deviation

	Mean ± SD	Median (Q1–Q3)	n (%)
Sex			
Male			34 (17.3%)
Female			163 (82.7%)
Age	86.8 ± 8.1	88.0 (82.0–92.0)	197
CCI	1.7 ± 1.2	1.0 (1.0–2.5)	197
Care need level	4.1 ± 0.9	4.0 (4.0–5.0)	197
1			3 (1.5%)
2			5 (2.5%)
3			36 (18.3%)
4			83 (42.1%)
5			70 (35.6%)
DSS	5.0 ± 1.3	5.0 (4.0–6.0)	197
3			25 (12.6%)
4			52 (26.4%)
5			47 (23.9%)
6			37 (18.8%)
7			36 (18.3%)
FOIS	5.5 ± 1.1	5.0 (5.0–7.0)	197
4			34 (17.3%)
5			81 (41.1%)
6			25 (12.7%)
7			57 (28.9%)
Number of teeth	11.3 ± 9.3	10.0 (2.0–19.0)	197
OHAT-j score	5.0 ± 2.8	5.0 (3.0–7.0)	197
Denture use			
With dentures			81 (41.1%)
Without dentures			116 (58.9%)
5-Defects score	3.3 ± 2.7	3.0 (1.0–6.0)	81 (41.1%)

**Table 2:** Multiple regression analysis of the association between dietary texture (FOIS) and denture wearing in older adults requiring long-term care (n = 197). Adjusted R² = 0.598, F=37.499, p-value=<0.001. Dependent variable: FOIS score (4 = pureed diet to 7 = regular diet). Independent variables: Age, sex (0 = male, 1 = female), CCI, care-need level (1 = mildest, 5 = most severe), DSS (3 = liquid aspiration level, 7 = within normal limits), number of teeth, OHAT-j score (0 = best oral health status, 16 = poorest oral health status), and denture use (0 = no, 1 = yes). CCI: Charlson comorbidity index; DSS: dysphagia severity scale; FOIS: functional oral intake scale; OHAT-j: oral health assessment tool (Japanese version)

Explanatory Variable	B	Standard Error	β	p-value	95% Confidence Interval	Variance Inflation Factor
Age	-0.020	0.007	-0.150	0.004	−0.034 to -0.006	1.285
Sex	<0.001	0.138	<0.001	0.998	−0.273 to 0.273	1.138
CCI	<0.001	0.043	<0.001	0.996	−0.085 to 0.085	1.023
Care need level	−0.230	0.061	−0.186	<0.001	−0.350 to −0.110	1.190
DSS	0.523	0.042	0.638	<0.001	0.441 to 0.605	1.248
Number of teeth	0.013	0.006	0.110	0.038	0.001 to 0.025	1.356
OHAT-j score	0.019	0.018	0.049	0.306	−0.017 to 0.054	1.090
Denture use	0.319	0.113	0.145	0.005	0.097 to 0.541	1.278

**Table 3:** Multiple regression analysis of the association between dietary texture (FOIS) and denture quality (5-Defects score) among denture-wearing older adults requiring long-term care (n = 81). Adjusted R² = 0.608, F=16.146, p-value=<0.001. Dependent variable: FOIS score. Independent variables: Age, sex (0 = male, 1 = female), CCI, care-need level, DSS, number of teeth, OHAT-j score, 5-Defects score (0 = best denture quality, 10 = poorest denture quality). CCI: Charlson comorbidity index; DSS: dysphagia severity scale; FOIS: functional oral intake scale; OHAT-j: oral health assessment tool (Japanese version)

Explanatory Variable	B	Standard Error	β	p-value	95% Confidence Interval	Variance Inflation Factor
Age	−0.005	0.011	−0.038	0.613	−0.027 to 0.016	1.143
Sex	-0.068	0.197	−0.026	0.730	−0.461 to 0.324	1.105
CCI	0.060	0.070	0.066	0.393	−0.079 to 0.199	1.173
Care-need level	−0.155	0.093	−0.129	0.099	−0.341 to 0.030	1.196
DSS	0.546	0.065	0.710	<0.001	0.417 to 0.675	1.409
Number of teeth	-0.003	0.011	−0.018	0.821	−0.025 to 0.020	1.225
OHAT-j score	0.035	0.028	0.103	0.214	−0.021 to 0.091	1.351
5-Defects score	−0.075	0.029	−0.198	0.012	−0.133 to −0.017	1.188

## Discussion

This cross-sectional study investigated the associations between diet texture and denture use, as well as between diet texture and objective denture quality assessment (5-Defects) among institutionalized older adults who required nursing care and could eat orally (FOIS 4-7). The results revealed that a high level of diet texture was associated with a good general health status, including a younger age and a lower care-need level, as well as high swallowing function, a greater number of teeth, and denture use. Furthermore, among denture-using older adults, high-quality dentures were associated with a high level of diet texture, similar to high swallowing function. To our knowledge, this is the first study to reveal that, beyond denture use itself, the objective quality of the dentures is associated with diet texture among dependent older adults. Denture use and improved denture quality are factors that can be enhanced through dental intervention and are readily applicable to older adults, even those for whom swallowing training to improve dietary texture is challenging.

The study participants were older adults with an average age of 86.8 years; 80% of them were females, approximately 70% had dementia, and the overall care dependency levels were high, with 80% requiring constant assistance. Furthermore, the average number of teeth was low. Despite the high demand for dentures, the denture use rate was approximately 40%. Thus, it is difficult to generalize the findings of this study directly to older adults with lower care needs. However, maintaining both general and oral health from middle age onward may be important for preventing future dependency. Previous studies on older adults requiring care have reported that longer time out of bed and higher ADL levels correlate with a higher level of diet texture, supporting the findings from this study that lower age and care-need level correlate with better diet texture [19]. Furthermore, while many studies have shown that swallowing function strongly influences diet modification, it has also been reported that swallowing function is associated with oral health status, oral function, diet texture, and malnutrition, suggesting that comprehensive evaluation and integrated management of these factors are crucial [20,21]. The results of this study, which showed that higher DSS scores were strongly associated with higher levels of diet texture, support these findings. Taira et al. also showed that the presence of dysphagia and denture use were more strongly associated with maintaining a high food texture level than age, ADL, or number of teeth, indicating the importance of occlusal support in maintaining diet texture for older adults requiring care [13]. Our findings support these results and further emphasize the impact of denture treatment, as improving denture quality through appropriate prosthodontic care may promote denture use and help maintain higher diet texture levels. Furthermore, while the swallowing function tends to be prioritized over the masticatory function when adjusting for diet texture, the masticatory function is a critical factor in determining diet texture modification and directly affects the enjoyment of eating.

Masticatory function is significantly influenced not only by the condition of the remaining teeth but also by denture quality, which affects denture-wearing status [22]. However, previous studies have mainly focused on the presence or absence of dentures, not sufficiently examined the denture quality by evaluating factors such as retention and stability. The findings showed that higher quality dentures were associated with a higher level of diet texture in addition to denture use, highlighting the importance of not only wearing dentures, as Taira et al. reported, but also their functional quality [13]. Although an association with diet texture was identified, the relatively weak strength of this relationship might be explained by the fact that the denture quality among denture wearers in this study was generally high. Well-fitted dentures remained stable in the oral cavity during mastication and swallowing, contributing to an improved QOL with or without cognitive decline, through the appropriate exertion of tongue pressure during swallowing and enhanced masticatory function [23-25]. Findings from this study also suggest the importance of objectively evaluating the denture quality. The Hummel et al. study used to evaluate denture quality in this research revealed that approximately 65% of the dentures examined had some defects, and only approximately one-third were considered good [18]. This finding suggests that many denture users are forced to use low-quality dentures, and their diet quality might be low [26]. Previous studies have identified cognitive decline and reduced oral motor function as major factors contributing to denture nonuse among older adults requiring care [27,28]. Mastication is an integrated oral movement; when oral function declines with aging, older adults might become less able to use dentures effectively during mastication, and this difficulty is exacerbated by the use of low-quality dentures [28]. Maintaining a high masticatory function is crucial for the enjoyment of eating and efficient nutritional intake. It is often difficult for older adults requiring long-term care to perform swallowing training aimed at improving diet texture due to cognitive and physical impairments [4]. Denture quality can be improved through dental intervention only by dentists, and the use of well-fitted dentures contributes to maintaining and improving diet texture. The findings of this study, which clarify this point, define the role of dentistry in home-visit care, specifically in denture treatment, maintenance of remaining teeth, and dysphagia rehabilitation, within a multidisciplinary team approach to providing meal support for older adults requiring care.

This study had several limitations. First, this was a retrospective, single-center, cross-sectional study with a limited number of participants; the causal relationship between denture quality and diet texture should be analyzed in a future study. Additionally, oral health status was assessed by multiple dentists. However, inter-rater reliability of the assessment method, as measured by the intraclass correlation coefficient, was high, suggesting a minimal impact of this factor [17, 29]. Second, the participants were limited to older adults requiring care who could consume all nutrition orally; those receiving supplemental nutrition and partially consuming it orally were excluded. Third, although we included the number of remaining teeth as a confounding factor, detailed denture information, such as type (complete denture/partial denture), unilateral use, placement site, and frequency of use, was not included in this study, leaving the impact of these factors on denture quality and diet texture unclear. Furthermore, precise cognitive function and psychosocial factors such as appetite, food preferences, social engagement, and motivation for daily living were not assessed, and the care-need level was used as a proxy for these factors. Additionally, factors such as whether a high-level diet texture was chosen despite the risk of pneumonia [30], or whether the preferences of the individual or family were considered, were not evaluated. Future research should address these limitations and incorporate quantitative assessments of oral function, evaluations of meal duration, and the level of assistance required during eating.

## Conclusions

Factors associated with a high level of diet texture among institutionalized older adults include a greater number of remaining teeth, younger age, lower care dependency, higher swallowing function, and denture use. Furthermore, the use of high-quality dentures was associated with better diet texture. The findings of this study suggest that prosthetic intervention to improve denture quality and ensure denture use among older adults requiring care is as important to support a high level of diet texture as maintaining the number of remaining teeth and providing dysphagia rehabilitation.
